# SPSmart: adapting population based SNP genotype databases for fast and comprehensive web access

**DOI:** 10.1186/1471-2105-9-428

**Published:** 2008-10-10

**Authors:** Jorge Amigo, Antonio Salas, Christopher Phillips, Ángel Carracedo

**Affiliations:** 1 Spanish National Genotyping Center (CeGen), Genomic Medicine Group, CIBERER, University of Santiago de Compostela, Galicia, Spain; 2 Forensic Genetics Unit, Institute of Legal Medicine, University of Santiago de Compostela, Galicia, Spain

## Abstract

**Background:**

In the last five years large online resources of human variability have appeared, notably HapMap, Perlegen and the CEPH foundation. These databases of genotypes with population information act as catalogues of human diversity, and are widely used as reference sources for population genetics studies. Although many useful conclusions may be extracted by querying databases individually, the lack of flexibility for combining data from within and between each database does not allow the calculation of key population variability statistics.

**Results:**

We have developed a novel tool for accessing and combining large-scale genomic databases of single nucleotide polymorphisms (SNPs) in widespread use in human population genetics: SPSmart (SNPs for Population Studies). A fast pipeline creates and maintains a data mart from the most commonly accessed databases of genotypes containing population information: data is mined, summarized into the standard statistical reference indices, and stored into a relational database that currently handles as many as 4 × 10^9 ^genotypes and that can be easily extended to new database initiatives. We have also built a web interface to the data mart that allows the browsing of underlying data indexed by population and the combining of populations, allowing intuitive and straightforward comparison of population groups. All the information served is optimized for web display, and most of the computations are already pre-processed in the data mart to speed up the data browsing and any computational treatment requested.

**Conclusion:**

In practice, SPSmart allows populations to be combined into user-defined groups, while multiple databases can be accessed and compared in a few simple steps from a single query. It performs the queries rapidly and gives straightforward graphical summaries of SNP population variability through visual inspection of allele frequencies outlined in standard pie-chart format. In addition, full numerical description of the data is output in statistical results panels that include common population genetics metrics such as heterozygosity, *Fst *and *In*.

## Background

The diverse nature of the major online SNP databases requires the researcher interested in population variability to query each in turn, to obtain allele frequencies, then to compile their own statistical indices for comparison of populations within and between databases. This task is made more difficult by the different data formats of the results given by each database, whose focus does not always address the needs of researchers interested in population variability, and in some cases it may be necessary to download large data segments to run locally a specific population based analysis.

Because the large-scale SNP data repositories are heterogeneous, and in response to our own need for a graphical browser for complex and extensive SNP data where this was lacking, we developed a system to summarize genotypes from multiple populations quickly and easily. The system uses a fast pre-processing pipeline able to work with any population based SNP database and can bring together disparate information into more informative summaries of variability, locus data and statistical metrics.

## Methods

### SNP resources

Many online databases that catalogue human variability provide population information about the samples studied, notably HapMap [1,2], Perlegen [3] and the CEPH foundation [4,5]. For instance, data from the CEPH Foundation collating genotypes generated from the human genome diversity panel (HGDP) gives one of the most valuable resources for human population studies in terms of geographic coverage and samples analyzed (1056 samples from 51 diverse populations), with recent contributions releasing major quantities of genotypes, e.g. the Stanford University CEPH-HGDP SNP genotyping initiative has yielded 650,000 SNP genotypes in 971 samples [6]. However, the data is accessible only as flat text files of limited use for many of the needs of population and evolutionary genetics studies. The Michigan University CEPH-HGDP SNP genotyping initiative has replicated in large part that of Stanford, so both databases overlap significantly in SNPs and samples genotyped. Therefore these databases cannot be considered as fully independent when carrying out population genetics studies.

In contrast to the Stanford and Michigan databases, the HapMap Phase II database contains an extensive amount of common genetic variation characterized in just four population samples. One of the main aims of HapMap Phase III was to extend the genotyping to a wider range of populations comprising SNP data of 1,115 individuals from 11 populations. The Perlegen database is also extensive in terms of SNP number but limited in terms of populations studied.

Some SNP repositories have web-sites that allow the downloading of SNP genotypes and locus information (chromosome position, linkage disequilibrium, etc.). However none permit the comparison and re-combination of multiple populations or the computation of population genetics indices. The SPSmart addresses this gap in possible analysis approaches by allowing the user to make specific searches of SNP lists in chromosomal regions and/or genes and to make comparisons of SNP variation within and between each of the databases outlined on Table 1. In particular the option to compare SNP variability across different databases provides a valuable system for initiating SNP based population genetics studies.

**Table 1:** Main characteristics of the SNP databases currently accessed by SPSmart

Database	SNPs	Populations	Web Address
HapMap Phase II	4,098,495	4	

HapMap Phase III	1,614,792	11	

Perlegen	1,580,349	3	

Stanford HGDP	660,918	52	

Michigan HGDP	525,910	33	

### Pre-processing the data

**Figure 1:**
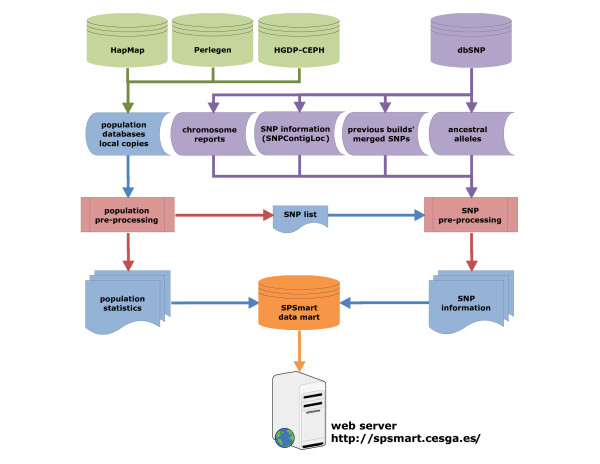
**Flowchart of processes implemented in SPSmart**. The underlying SPSmart processing engine is capable of dealing with virtually any database that contains genotypes grouped by populations. Any dataset is summarized into common populational statistical indexes, and then combined with dbSNP additional information in order to improve the online data browsing experience.

A common characteristic of the most widely accessed human population databases is infrequent or unpredictable update cycles. To remove the need of the user to check for updates we have implemented a fast pre-processing pipeline, able to work with any given SNP genotyping database that reports multiple populations, which can summarize information into the most useful statistical indices (allele frequencies, heterozygosity, *Fst *[7-9] and *In *[10]). Scripts generate a data mart from the pre-processed data of the most recent database build in multiple flat files and merges these with the latest dbSNP build (mid 2008: #129) to acquire additional SNP information such as chromosome, position, validation status, gene, reference allele, and ancestral allele derived from the Chimpanzee genome. Although each query would normally demand its own processing resources, pre-processing the data solves the major computing issues, so serving all these calculations through the web was the next logical step as shown on the workflow described on Figure 1.

All the SNP repositories that have been processed have their raw data freely available for bulk downloading. Their genotypes are compressed in plain-text files arranged in tables, differing only in the structure of those tables: Hapmap, Perlegen and the Stanford CEPH present their data in a SNP per row basis, with the samples in columns, while the Michigan CEPH data is arranged following the structure format (that is a sample in each couple of rows, with each SNP's allele 1 and allele 2 contained on the first and second sample line respectively.

The pre-processing engine has three major aims: (i) to rewrite the data into a more appropriate format for population combinations, (ii) to build all the possible summaries that may be requested by populations, and (iii) to merge the genotype data with dbSNP information. The output of the population pre-processing of any repository is a SNP list containing all the SNPs found in the database and files containing all the calculated statistical indexes per SNP and per population. The SNP list is used to retrieve additional dbSNP information through a collateral pre-processing engine, aiming to enrich the data mart. Placing the data into a relational database allows quick presenting of these pre-calculated results through the web interface, so a combination of those summaries for the requested population combinations is all that is required. As the major population groups can be expected to be queried often their combinations are pre-processed, hence statistical parameters of the populations that constitute the group are pre-calculated and stored too.

Including a new dataset is fairly straightforward: the format of the new dataset is analysed and, if needed, the reading module of the population pre-processing script is adapted. Once the data is read, the data is internally structured in identical fashion to the other datasets and subsequent pre-processing is executed in the same way. Updating incorporated datasets is easier still since no script adaptation is required, just a new pre-processing run that takes from several minutes to a few hours depending on data size.

### Programming languages

In order to satisfy the predicted needs demanded by SPSmart, a variety of programming languages were used in development. Perl was selected for all the pre-processing scripts, as it is recognized as one of the fastest programming languages for text-processing. The optimized regular expressions engine of Perl allows fast and reliable digesting of flat text files, so the resulting scripts are very powerful but undemanding in terms of resource consumption. To access the pre-calculated data mart and for presenting the data on each client, allowing user interaction through a web browser, the combination of PHP, MySQL and HTML was chosen, with due regard for common web standards such as CSS and XHTML, maximizing independence across different browsers. In addition Javascript was used to facilitate user input on the search section, to hide and show the results tabs, and for some minor design details. In combination the languages used produced a final web interface capable of rapid presentation of results while generating light pages for low bandwidth users.

## Results

All the data is stored in a relational database such that each available genotyping dataset has a table of SNPs with their descriptive information, a table of genes and the SNPs present in them, population data for listing purposes, and a table per population and per population group containing their summarized statistics. We have also built a web interface to allow browsing the data mart and to structure queries by populations where users can select any combination of populations within a database to obtain sets of comparative data and statistical metrics.

Populations can be combined on the advanced search page with the possibility of comparing up to five different population combinations. The user should be aware that the sampling approaches and sample sizes of each repository are different and this must be taken into account when inter-population comparison are made, even if populations carry identical descriptions.

### Tool usage

A SNP search can be performed by entering a list of SNPs, defining a chromosome region, or entering a gene name. The query reports results in a paginated manner comprising a frequencies tab for visual inspection of allele frequencies plus a statistics tab for a detailed and downloadable table containing statistical information for the queried SNPs for the defined population groupings.

**Figure 2:**
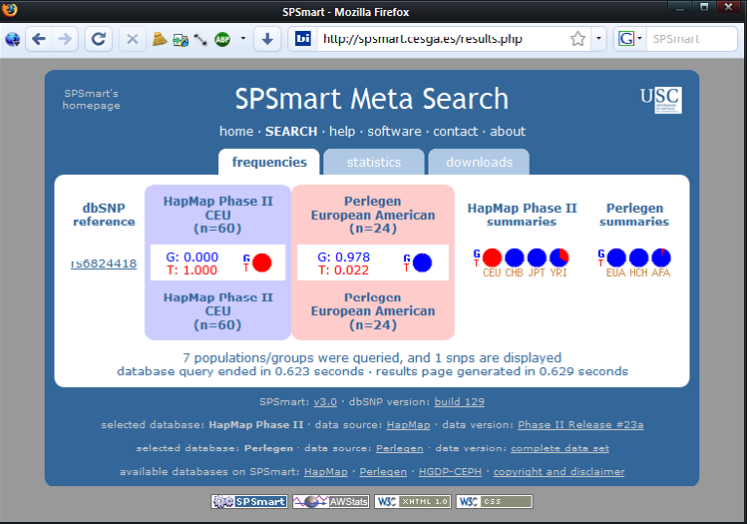
**Finding discrepancies among databases**. SNP rs6824418 data from Perlegen and HapMap indicating discrepant allele frequency estimates for populations EUA and CEU (European American and CEPH European respectively).

**Figure 3:**
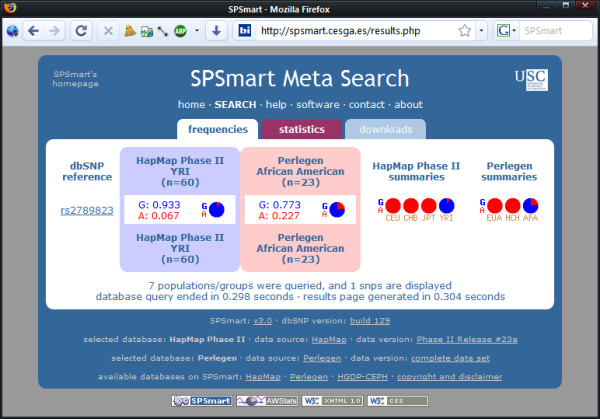
**Comparing similar populations in different databases**. SNP rs2789823 data from Perlegen and HapMap illustrating a fixed difference SNP that shows the degree of European:African admixture in the African American population sample of Perlegen (AFA) compared to the HapMap African population: the Yoruba of Ibadan, Nigeria (YRI).

**Figure 4:**
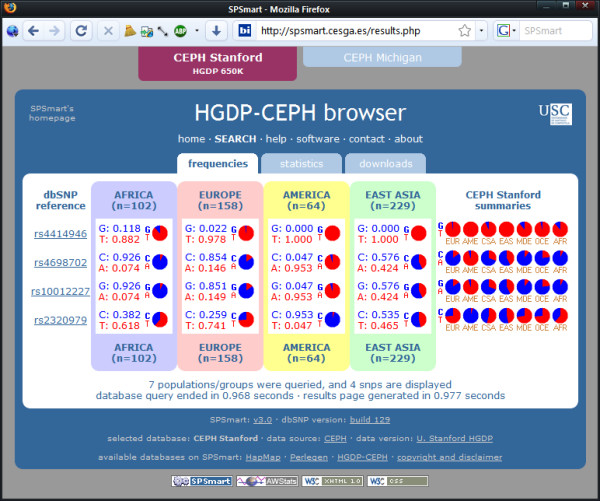
**Inspecting a chromosome region**. Using a chromosome region search to find an alternative SNP marker with improved quality flanking sequence in the same linkage disequilibrium block (rs10012227 as a better substitute for rs4698702).

Figures 2, 3 and 4 show three examples of queries that illustrate comparisons between databases and populations that illustrate the flexibility of comparing data from different sources with unified queries. Firstly (Figure 2), a comparison of population variability reported by Perlegen and Hapmap for SNP rs6824418 highlights a major discrepancy between these databases for Europeans: listed as an allele frequency of 0.065 in Perlegen (EUA) and 1 in Hapmap (CEU). Secondly (Figure 3), analysis of a fixed difference SNP such as rs2789823 provides a means to gauge European-African admixture in African Americans by comparing the reported variability in the Perlegen African American sample (AFA) with that of the Hapmap African sample of the Yoruba of Ibadan, Nigeria (YRI). Lastly (Figure 4), a SNP collected as a Native American informative ancestry marker (rs4698702) that has poor quality flanking sequence and cannot readily be genotyped may be substituted by performing a region search on chromosome 4 from 18230000 to 18250000 and locating a better alternative SNP (rs10012227) with a near identical frequency distribution due to association.

### Updating frequency

The frequency of updates of the available datasets and how these are incorporated in SPSmart depends largely on the databases themselves. If they are updated at their origin SPSmart can refresh the contents within a day of notification, however the main reference database of HapMap changes about twice a year, while Perlegen and CEPH databases are not expected to change at all. In addition, the SPSmart is designed in a way that allows easy implementation of new functionality. For instance, it is straightforward to implement new statistical indices as well as new filtering properties in response to user demands or changes in statistical approaches to population analysis reported in the literature.

## Discussion

The major novelty of SPSmart is the ability to combine populations from within a database and to compare populations between different databases, then from both of these operations derive key population variability statistics.

The SPSmart engine  provides several aids for population genetics and complex disease association studies; areas of research that can both be reliant on comparing SNP variability between populations. The SPSmart engine has successfully processed, and is currently running, a range of data sets encompassing: HapMap release #23a, the Stanford University and Michigan University CEPH-HGDP panels, and the Perlegen SNP data set. We aim to include any major new data sets that collate human SNP variability and implement an extended menu of statistical indices to further aid the population geneticist trying to make sense of the growing wealth of online SNP data.

## Conclusion

There are a very large number of autosomal SNP genotypes freely available in the literature and databases. Each database resource presents its own storage procedures and formats and therefore it is difficult for a researcher to use and combine the data from these resources. To our knowledge, this is the first web tool that allows the combination of different datasets of human SNPs and population groups, and to compute statistical indices of interest for medical and population genetics investigations.

## Availability and requirements

• Project name: SPSmart

• Project home page: 

• Operating system: Platform independent.

• Programming languages: Perl, PHP, SQL, HTML and JavaScript.

• Type of access: this web tool is freely available for non-commercial use.

## Authors' contributions

JA carried out the design, programming and implementation of the software, and drafted the manuscript. AS, CP, and AC participated in the design of the software and the databases selection, and helped to draft the manuscript. All authors read and approved the final manuscript.
